# Care Delivery for Patients with Leiomyomas: Failures, Real-Life Experiences, Analysis of Barriers, and Proposed Restorative Remedies

**DOI:** 10.1089/heq.2022.0116

**Published:** 2023-08-25

**Authors:** Veronica T. Lerner, Nicole M. Donnellan, Mathew T. Siedhoff, Mireille D. Truong, Cara R. King

**Affiliations:** ^1^ Department of Obstetrics & Gynecology, Lenox Hill Hospital, Northwell Health, New York, New York, USA.; ^2^ Department of Obstetrics, Gynecology and Reproductive Sciences, UPMC Magee-Women's Hospital, University of Pittsburgh School of Medicine, Pittsburgh, Pennsylvania, USA.; ^3^ Division of Minimally Invasive Gynecologic Surgery, Department of Obstetrics & Gynecology, Cedars-Sinai Medical Center, Los Angeles, California, USA.; ^4^ Section of Minimally Invasive Gynecologic Surgery, Obstetrics, Gynecology, and Women's Health Institute, Cleveland Clinic, Cleveland, Ohio, USA.

**Keywords:** leiomyomas, delivery of care, uterine artery embolization, coordination of care

## Abstract

In this narrative review, we describe historical and contemporary influences that prevent patients with fibroids from getting appropriate medical care. Using patient stories as examples, we highlight how misogyny on all levels hurts patients and prevents medical teams from doing their best. Importantly, inequity and disparities result in massive gaps in care delivery. We suggest that we, as gynecologists and surgeons, must join public discourse on this topic to highlight the inadequacies of care delivery and the reasons behind it, suggest potential solutions, and join patients and communities in formulating and implementing remedies.

## In the Trenches

“I first met Mrs. A, a frightened 49-year-old woman, when her son brought her to the emergency room after a syncopal episode. She was found to be in septic shock from an infected uterus after undergoing a uterine fibroid embolization (UFE) one month prior. I took to her the operating room to do an emergent abdominal hysterectomy where she was found to have a 32-week-sized uterus with several large pedunculated fibroids, one of which was an infected necrotic 10-centimeter pedunculated myoma with purulent matter leaking into the abdomen. After surgery, she spent several days in the intensive care unit and was discharged from the hospital a week later. Her recovery was long, but she was able to recover and return to her activities of daily living and her job as a manager in a bank.

Mrs. A was under the care of one of the community gynecologists. She was trying to avoid surgery. Although her gynecologist (private practice, mostly office setting, rare open hysterectomies, and no minimally invasive gynecologic surgery [MIGS] experience) counseled her that she was not a good candidate for UFE because of the large fibroid burden, high failure rate, and greater risk, the patient self-referred to a local ‘fibroid center’ and underwent a UFE. After her procedure, she started to develop worsening pain and malaise. She called her interventional radiologist several times to report her symptoms. He reassured her that this was expected during her recovery and did not evaluate her.

I called the interventional radiologist. While concerned with her clinical course, he did not even consider the appropriateness of UFE in her case. Several months later, I heard from Mrs. A's attorney. The patient was seeking restorative justice, wanted an acknowledgment of what happened to her, and wanted to ensure that the same did not happen to other women. Her attorney declined to take her case due to a ‘low anticipated payout.’ Since this interventional radiologist was in private practice, there was no ‘quality and safety’ entity to which to report him.

I discussed the case with my hospital's risk management department, but they had no recommendations other than reporting him to the state department of health and were reticent to become involved as none of our institution's physicians were involved in the decision to perform UFE. From the patient's perspective, none of these attempts led to anything meaningful for her in the way of justice.”

This narrative by one of the authors (V.T.L.) of Mrs. A's case is not in isolation. We often share our “war stories,” such as postmenopausal women with postmenopausal bleeding without endometrial sampling, women planning to undergo assisted reproductive technology (ART) treatments, and young women with very high fibroid burden desiring fertility getting UFE. On the contrary, in many of our surgical communities, we have wonderful interventional radiology (IR) colleagues who work collaboratively with us. Together, we build service lines, staff joint IR/MIGS clinics, review difficult cases in treatment planning conferences, and engage in quality improvement initiatives to make sure patients get access to the information they need to make informed decisions. Our IR colleagues save lives in gynecologic emergencies where surgery is not the best option.

Just as we have concerns about inadequate care by interventional radiologists and their incentive and bias due to favorable reimbursement in an outpatient setting, we are equally disappointed with gynecologic surgeons who do not meet the current standard of care. Examples of failure to meet gynecologic surgical standards of care for leiomyomas include the following: choosing the open surgery route while the majority of hysterectomies and myomectomies can be done via the minimally invasive surgery (MIS) route, unindicated surgery such as removing asymptomatic and small subserosal fibroids because those surgeries are easy, and failing to remove fibroids in more difficult locations. This, however, is not surprising given that Current Procedural Terminology codes do not incentivize the MIS approach, and are the same regardless of case complexity, while high-complexity MIS cases involve more work and risk.

Although the MIS approach is feasible in more than 90% of cases, many surgeons still operate through open incisions because of a lack of training and do not refer patients to those who have this expertise.^1^ Gynecologic surgeons also may advertise directly to patients or avoid caring for complications or sequelae of procedures they perform by seeking privileges in different hospitals.

Another aggravating gap in care that gynecologic surgeons often deal with when seeing patients referred for fibroid surgery is the lack of discussion of nonsurgical alternatives by referring providers who tell patients surgery is “the only option,” or being told that they are not candidates for surgery without more in-depth consideration as to why and without seeing the big picture and nuances specific to each patient. Being informed about how UFE works and how it applies to them often brings enormous relief for patients, regardless of the final decision about the treatment they make. For every case of Mrs. A, we can come up with many stories of fibroid mismanagement in the gynecologic community.

Disparities and inequality amplify all of those gaps. Patients of color and minorized and marginalized people suffer from higher disease burdens and have less access to high-quality medical and surgical care.^2–5^ Those are examples of what goes wrong with the delivery of care in all aspects of medicine, not just fibroids, gynecology, and surgery.

While the topic of why patients are not getting good care regardless of the person, disease, and specialty is an enormously complicated one, sweeping it under the rug because “it is complicated,” “too broken to fix,” and “impossible to get right” only worsens the extent of moral injury that results in clinicians' post-traumatic stress disorder and burnout. We, gynecologists and surgeons, need to own our problems and get over our tendency to bear the burden of not being able to do our best because of the societal and “system's” problems. We need to get our narrative out to the public. By doing that, we could find common ground, connect with each other and many advocacy entities, and convey meaning and purpose.

Our professional struggles and experiences, if heard by those around us, can unify us and help us act, even if this action does not result in an immediate meaningful change. While health care professionals can have this dialogue on their terms on social media, online blogs, lay publications, and sociology realms, these conversations are not commonplace in academic discourse in specialty journals.^1,6^

The objective of this article is to dissect problems in the delivery of care to women from the perspective of MIG surgeons using UFE as an example, point to deep-rooted causes of those problems, and suggest potential solutions and future directions that individuals and organizations can take to address them.

### Historical perspective, unmet need, devaluation of women's health, and systemic discrimination

When UFE emerged as a therapeutic option for fibroids in the 1990s, comparison with surgery became an important issue. At the time, there was no standardized way to assess outcomes from a patient's perspective. Unfortunately, common gynecologic conditions, such as fibroids and endometriosis have a major negative impact on the quality of life of millions of women and could lead to serious morbidity, did not have standardized measurement tools. Without such tools, quality research of treatment options was not possible, and this continues to be problematic today.^7^

Work by Carlson and colleagues focused on generic measures, but tools that assessed patients' perspectives did not exist.^8–10^ As a result, when Spies and his IR team started working on measuring UFE outcomes, they felt that they needed to create their own quality-of-life questionnaire for the public domain because of the lack of outcome assessment tools from the patient's perspective in the gynecology literature.^11^ Spies' work resulted in UFS-QOL. As an interventional radiologist, Spies was revolutionary in advocating for incorporating patients' take on their treatment. He worked with a group of pharmacodynamic experts funded by an IR research foundation and they created the questionnaire that is now the current standard for assessing outcomes from all fibroid interventions (pers. comm.).

This historical perspective demonstrates a fundamental lack of focus on what is important to women that has persisted in medicine for centuries. Unfortunately, this thinking is still ingrained in our culture. This systemic and long-standing devaluation of women, misogyny, and sexism explains why patients and patient advocacy groups keep asking us “How can this be that we are still not listening to women?” Women are still being told that painful and heavy periods are “normal” and they just “need to deal with it.” Diagnosis of endometriosis, for example, is thought to be delayed by 4–11 years.^12^

Likewise, we think that a large proportion of women with symptomatic fibroids are delayed in getting care or are not able to access it at all, although that has not been documented as accurately for this condition.^13,14^ Unfortunately, this leads not only to the undertreatment of these common conditions but also to the lack of resource allocation for research on women's health.

#### Direct restorative remedy #1: go public

Acknowledge past and current discrimination in women's health publicly by bringing patients' and clinicians'stories of how the medical system and our society ignore common medical conditions such as fibroids. While social media platforms have become one way to draw attention to the topic and for clinicians and patients to be heard, we think that professional societies and mainstream medical journals need to create truth and reconsolidation entities. This will create spaces necessary to have these conversations in a wide array of settings and will deliver this information to the public. It will also allow clinicians to partner with patients, community, and equity advocacy groups to build effective medical teams and diversify leadership entities.

## The Evidence: Knowns and Unknowns

### Lack of an integrated approach and meaningful patient-centered shared decision-making

Let us take a closer look at the evolution of UFE efficacy data. Excitement about UFE started in 1995 when Ravina et al. published the first case series of UFE for the management of fibroids,^15^ followed by a series of publications from the Fibroid Registry For Outcomes Data (FIBROID).^16–18^ The FIBROID was created in 1999 and prospectively enrolled more than 3000 patients. Broad support for it in the IR community reflects the dedication many interventional radiologists have to advance patient care and understanding of outcomes. One of the last landmark publications from this registry noted a lasting symptomatic relief after UFE but also found the risk of hysterectomy, myomectomy, and repeat UFE to be 9.79%, 2.82%, and 1.83%, respectively, 3 years after the procedure—significant long-term failure rates that are not consistently addressed in day-to-day clinical conversations.^18^

The HOPEFUL study, a multicenter retrospective cohort study from the United Kingdom, followed the steps of the FIBROID registry and reported on outcomes and efficacy in a minimum of 2 years of follow-up (972 hysterectomies and 762 UFE cases).^19^ Fewer serious complications were noted in the UFE group with more women reporting symptomatic relief, and more women stating that they would recommend their treatment to a friend. Up to 23% of women in the UFE group received more than one treatment, and 11.2% had a hysterectomy. In younger women with a higher risk of recurrence, UFE was not cost-effective.

The EMMY study (The Uterine Artery Embolization in the Treatment of Symptomatic Uterine Fibroids Tumors-EMbolization vs. HysterectoMY) examined long-term, 10-year outcomes.^20^ This European randomized trial demonstrated that of 156 patients in the original cohort, 84% reported similar patient satisfaction rates and quality-of-life metrics in both UFE and hysterectomy groups. However, 35% (28 out of 81 patients) had hysterectomies for recurrence after UFE, in addition to other surgical reinterventions.^20^ Similar outcomes are noted in IR literature.^21^

While these data are not perfect, we can surely come up with a way to convey it along with the limitations and context to our patients. One may ask then, why then when we ask patients why embolization failed, what their recollection was of what they were told about failure risk before the procedure, responses range from “nothing” to “I was told I might need to do it again.” Similarly, the same could be said about the patient's recall of surgical risk discussed at the preoperative visit. The subject matter of conveying medical information that patients need to make decisions, especially since we know that patient recall is minimal long term even after extensive counseling for high-stake procedures is complex.^22^ While there have been many articles and teaching aids written on UFE and fibroid management, what is lacking are granular implementation pathways on how to convey this information to patients.

Just like Basic Cardiac Life Support certification is mandated by hospitals, the step 3 clinical skills examination and Fundamentals of Laparoscopic Surgery examination are mandatory to become board certified, postpartum hemorrhage team training is required by the Joint Commission to occur on labor and delivery units, and surgical time-outs are videotaped by hospitals to assure compliance with safety checklists—same can be done with any other clinical activity. Many medical schools and residencies, frustrated with the Accreditation Council for Graduate Medical Education milestones (which set learning agenda for doctors in training) that lack objective practical nuance needed for learning assessment, and are ridden with bias in how they are implemented, are turning to entrustable patient activities, along the lines of what is being used in Europe for competency-based certification and credentialing.

Competency in shared decision-making and the tools needed to accomplish it can also be mandated. This would require a major culture change, but we have the tools to accomplish it if desired.

#### Direct restorative remedy #2: fix information sharing and communication processes

Acknowledge that many patients are not getting adequate information during counseling, are not given the information they need to make decisions, and are not supported in their decision-making. Invest in national “patient-centered education” infrastructure. Make it mandatory for the federal government to invest in professional societies, hospitals, communities, patient advocacy groups, and individual practitioners to create and implement high-quality decision aids, tools, and ways of relaying information that would help patients understand treatments and how those treatments apply to them. Encourage health professionals to embrace a growth mindset, advocate for clinical competency assessment, and monitor clinician adherence.

### Efficacy and safety of UFE, comparisons with surgical options, and failure of research infrastructure to deliver meaningful information to clinicians and patients

While long-term surgical retreatment rates for recurrence after UFE must be shared with patients who are not interested in fertility or to whom uterine conservation is not a priority, it is worth pointing out that the decision for surgery versus UFE is more nuanced than just deciding to have an operation. While hysterectomy results in definitive management, it does not make sense to compare hysterectomy outcomes with UFE in terms of long-term relief as UFE will always have a retreatment risk. Most patients are deciding if they want to “get it over with once and for all” and have a hysterectomy or take a chance that UFE would work if properly selected with the main goal of avoiding surgery.

A meaningful comparison would be to look at surgical retreatment rates after UFE versus myomectomy for those patients concerned with keeping their uterus for reasons other than fertility. Here we need to consider surgical morbidity after a myomectomy (especially if not done minimally invasively). While several publications show similar retreatment rates in the long term,^23–25^ the recent multicenter study from the United Kingdom, the FEMME trial (A Randomized Trial of Treating Fibroids with Either Embolization of Myomectomy to Measure the Effect on Quality of Life Among Women Wishing to Avoid Hysterectomy) examined outcomes of 254 women.

While the study was not able to recruit the planned number of patients after 2 years, both groups improved significantly, but health-related quality-of-life scores were minimally higher in the myomectomy group, complications were the same in both groups, and more surgical reinterventions occurred in the UFE group. The myomectomy route was not reported or analyzed.

Regarding uterine conservation, unfortunately, published data on repeat myomectomies are not granular enough to be useful clinically in terms of surgical morbidity in primary and repeat myomectomies and reproductive outcomes. We have encountered patients who had myomectomies before ART therapies such as *in vitro* fertilization (IVF) to maximize implantation rates but ended up with failed embryo transfers despite having favorable prognostic factors. At times, fluid is seen in the cavity, and defects are noted to be similar to a cesarean section niche, suggestive of uterine factor. Would these patients do better with UFE if they had frozen euploid embryos from IVF treatments and just needed fibroid shrinkage of small fibroid burden, especially if they had a prior myomectomy? We do not know the answer to this and many related nuanced questions.

Deeper questions remain unanswered concerning long-term outcomes and patient selection. As UFE became more common, several studies comparing UFE and surgery were conducted by Refs.^26–33^ A 2014 Cochrane systematic review included data from 7 randomized controlled trials (RCTs) with 793 women.^34^ Based on limited evidence, authors concluded that there was no difference in satisfaction rates between the surgery and UFE groups for up to a 5-year follow-up, but UFE was associated with a higher rate of minor complications and a higher risk of subsequent operation, quoted as15–32% within 2 years.^34^ Systematic reviews and meta-analyses continue to be published based on smaller limited studies.^34–39^

A robust comparative effectiveness review on the management of fibroids was published in 2017 and funded by the Agency for Healthcare Research and Quality. The authors firmly stated that “there is insufficient evidence for women to choose one intervention over another based on individual characteristics” due to a lack of power to examine subgroups for outcomes.^37^

Another impressive research effort currently in progress is Comparing Options for Management: Patient-centered Results for Uterine Fibroids (COMPARE-UF), a prospective multicenter fibroid registry in the United States.^40^ It is the largest federally funded outcome study ever, planned to enroll 10,000 patients who had procedures to treat fibroids. In contrast to prior simplistic and limited comparative studies, the authors planned to report on the efficacy of all routes of hysterectomy and myomectomy, endometrial ablation, radiofrequency ablation (RFA), UFE, magnetic resonance imaging-guided focused ultrasound (MRgFUS), progestin-releasing intrauterine device and to disseminate it to stakeholders.^40^ Currently, more than 3000 women are enrolled, and the plan is to follow the cohort over 2 years.^40^ Unfortunately, even this massive undertaking ran into logistical problems of research infrastructure, volume, and ability to get a representative sample, and, as a result, the number of women getting UFE was relatively small (pers. comm.).

Several articles describing women's experience with treatments, and comparisons in quality-of-life improvements between myomectomy and hysterectomy, including route, have recently been published, but long-term comparisons of surgery with UFE are not yet in print.^41,42^ We hope to see the publications from this landmark study shortly and to learn more about the difficulties of trying to do outcome-based research on a large scale in the United States in women's health, which lacks data collection and tracking researchers need to understand complex conditions affecting many.

Finally, while patient satisfaction and quality-of-life measures are very important, other parameters need to be captured such as uterine volume and fibroid burden (number, size, location). Given the inclusion/exclusion criteria in UFE studies and the variability of fibroids and the symptoms they cause, even randomized control trial data may be difficult to apply to individual patients in the current format.

UFE results in ischemic degeneration from selective catheterization of uterine arteries under fluoroscopic guidance via injection of embolic agents. Immediate procedural risks such as groin hematomas, allergic reactions, arterial thrombosis, and pseudoaneurysms are deemed to be low (<5%).^43^ However, other sequelae such as nonselective embolization have been associated with more serious yet still rare events such as dyspareunia and sexual dysfunction,^44^ vaginal, buttock, and subcutaneous tissue necrosis, and chronic pain.^3^

Given the steady increase in UFE procedures performed over the last two decades, outcome data are limited to registries and population-level studies and administrative databases similar to those that exist to describe surgical outcomes for fibroid surgeries^38,45^ do not exist for UFE. As a result, counseling patients based on outcomes with limited comprehensive and reliable sources of evidence is challenging. For example, the largest study to date was published in 2002, which included a single-center registry consisting of 400 patients who were prospectively followed for a minimum of 3 months after UFE.^46^ The overall complication rate was 8.5%, of which 1.25% was considered to be serious complications and included emergent hysterectomy, undetected leiomyosarcoma, pulmonary emboli, arterial thrombosis, leiomyoma passage associated with infection, and hemorrhage, and readmissions, with overall morbidity of 5%.^46^

While this study provides a snapshot of complication trends in 2002 for those 400 patients, limited reporting of UFE outcomes limits our ability to understand more contemporary trends. Another important factor to consider is the type of complications that occurs with fibroids based on size, number, and location. While we might be able to discuss specific RCTs with carefully selected patients, applying those findings in the “real world” is challenging as fibroids have such vast diversity in character and clinical presentation.

Published literature on complications to date is limited to case series^47,48^ and Manufacturer and User Facility Device Experience database reports, all of which lack a denominator, as well as meta-analyses that have their inherent limitations.^49^ While we inform patients about expected postprocedural pain and postembolization syndrome, counseling may not always include a discussion of prolonged vaginal discharge in up to 20% of patients,^17^ failure to complete procedure bilaterally reported as high as 5%,^50^ and myoma expulsion that ranges from 1.7% to 50%.^51^ Some of these complications require surgical intervention and may occur months to years following the procedure.^51^ Reported serious events also include perforated pyomyomas, pelvic abscesses, hematometra, fistulas, bowel obstruction, bowel perforation, uterine necrosis, uterine rupture, placenta increta, sepsis, pulmonary emboli, myocardial infarction, emergency surgery, and death.^49,52–58^

Of course, these are exceedingly rare and it is not possible to list all complications for procedural interventions, and serious complications are well-established risks of major surgery.^59,60^

Adequate counseling must also include a discussion comparing complications of UFE with those of alternative treatment options. Here, too, we lack the data we need to have meaningful discussions. Numerous studies that have examined this topic are outdated as they largely include open surgery for hysterectomy and myomectomy procedures. In an era where most benign gynecologic surgeries have shifted from open to MIGS routes, it is important to consider the changing national and local surgical landscape when counseling patients. In surgical centers where open surgery rates are low, high-volume surgeons are trained in MIS, and enhanced recovery protocols are in place, risk–benefit analysis of surgery compared with UFE is likely to differ from prior comparative studies with high open surgery rates.^30^

Just like UFE outcomes improve as procedural details were finessed over the first decade after its introduction, the formula for risk calculation has changed for surgery as well, but it is not reflected in the current literature, thus limiting providers in their efforts to perform the most appropriate informed counseling and consent when discussing appropriate treatment modalities for symptomatic fibroids.

Deficiencies in outcomes research are not unique to UFE. Surgical outcomes are in the same boat. While many of us keep our own outcome data to monitor our surgical performance,^1^ very few MIGS divisions who can afford an outcome nurse do so for their surgeon on a local level. We keep asking ourselves, “Why are we not reporting granular data for all surgeries and procedures on a national level?” Among many benefits of such an approach would be a way to help surgeons monitor their skills on an individual level in a standardized rather than self-reported way. On a larger scale, the scientific community and society as a whole will have a better understanding of diseases and treatments for the allocation of resources.

One of the main reasons why research is lacking in very common conditions that affect women such as fibroids and endometriosis affecting millions of women is because it is vastly underfunded by the NIH.^61^ A popular joke on social media to reflect on this notion is: “If men were suffering 10 days out of each month with horrible pain and bleeding that prevented them from functioning, having sex and reproducing, this a national emergency would have been declared long ago and we would have a cure for fibroids.” While we are debating on how to monitor surgical outcomes and how inaccurate reporting of them is, women are not living “well.”

While we complain about the lack of granular data even on a local level, it would take one research nurse to monitor surgical outcomes for a large MIGS division in a single hospital. Dr. Louse King writes about the sequelae of meaningful standardized surgical outcome tracking in her essay, describing what happens to the patients as result.^1^

#### Direct restorative remedy #3: mandate an increase in research funding

Demand a complete overhaul in outcomes research. Acknowledge and specify the extent to which we lack the resources to do so. Expose weaknesses, lack of nuance, and gaps in current research infrastructure that prevent delivery of studies meaningful to patients and clinicians. State what it would take to create and maintain outcomes research infrastructure on national, regional, local, and institutional levels.

### Failure to address fertility, menopause and sexual function, risk of malignancy, and the need for comprehensive gynecologic and medical preprocedure evaluation

#### Fertility

The concern for patients desiring future fertility is that the direct ischemic insult to the uterus and ovaries from embolization could increase the risk of ovarian failure, decrease ovarian reserve, and impair uterine implantation. If blood flow from ovarian arteries to fibroids is noted postprocedure, ovarian blood supply is embolized due to the concern that collateral blood supply would increase failure risk.^62^

Literature to inform patients about post-UFE conception rates is limited.^63^ A recent metanalysis approximated fertility rates after UFE as 29%^64^ and 38%,^63^ which are lower than the assumed rates for the age groups reported. The only RCT to date is a study by Mara et al. published over a decade ago, which sought to compare outcomes of UFE versus myomectomy in women desiring to conceive.^30^ In this trial, UFE patients had significantly lower rates of pregnancy than those in the myomectomy group (50% vs. 78%), and the relative risk of infertility after UFE was more than twice of those who underwent a myomectomy (RR=2.22; 95% CI 1.11–4.44). Although small, the majority of myomectomies were laparoscopic, which again supports the evolution of risk and benefit calculations in the early era of MIGS.

One of the critiques of the study design is that during the follow-up, patients had a secondary procedure (myomectomy) performed in approximately a third of UFE patients per study protocol in cases where fibroids did not shrink after UFE or if they were larger than 5 cm. As a result, more patients in the UFE arm ended up with a myomectomy during follow-up, which potentially lowered their fertility rate. Based on this one study, the Cochrane review in 2014, authors warned that those findings should be interpreted with caution.^65^ It is unlikely that another randomized trial looking at fertility outcomes after UFE versus myomectomy would be possible shortly.

Equally important is addressing contraceptive management after UFE for those patients who may not wish to conceive. Data are limited regarding outcomes in women who were either ambivalent about childbearing or those who ended up with unplanned pregnancies that they decided to continue. There are reports of higher miscarriage rates in the UFE pregnancies (35.2% vs. 16.5%, OR 2.8 with 95% CI 2.0–3.8).^66^ No recent work has been done to further clarify higher risks of abnormal placentation, preterm labor, postpartum hemorrhage, and cesarean delivery.^66–68^ Is embolized uterine tissue biologically different from nonembolized? What is the ischemic effect of embolization on myometrium in women who get spontaneously pregnant or those who undergo ART? We do not have clear answers.

Furthermore, there is limited published information regarding the role of contraceptive counseling or referral for such counseling during a consultation with an interventional radiologist, which is paramount for those undergoing UFE and not desiring future fertility.

#### Premature menopause and sexual function

Women facing hysterectomies commonly have concerns with their sexual function. The risk of an earlier onset of menopause thus becomes a common topic of discussion at the time of decision for surgery. In the evaluation of post-UFE amenorrhea along with Anti-Mullerian Hormone, follicle-stimulating hormone, and antral follicle count, early studies have shown ovarian failure rates that ranged from 6% to 14%.^67–70^ Decrease in ovarian reserve after UFE is age dependent with older women experiencing greater decline and younger populations experiencing more transient changes, comparable with the effect of surgery.^71,72^ How clinically meaningful this risk of loss of ovarian reserve in younger women trying to conceive is not known. While concerns about the impact of hysterectomy on ovarian function have been raised over the last decade, evidence from several randomized control trials showed that hysterectomy results in comparable rates of ovarian failure, and women closer to menopause are at higher risk.^73,74^

Little is known about sexual function after UFE. A 2017 prospective descriptive study noted effects similar to that described after hysterectomy: as symptoms are treated, sexual function improves.^75^ In comparison, this has been extensively looked at for patients having hysterectomies; from the patient's perspective, a hysterectomy seems similar to a more “invasive” procedure that takes the fertility symbol (the womb) away from them and as a result, patients might think that UFE would be less damaging to their sex lives although no literature exists to the best of our knowledge on this topic about UFE.^76^ Aside from this specific patient concern, several studies have shown that sexual function actually improves after a hysterectomy.^77–80^

#### Malignancy

Another concern at the time of UFE is unrecognized cancer risk and missing occult cancer (cervical, endometrial, and uterine), despite standard-of-care preoperative workup and especially if a patient has not been evaluated or referred to a gynecologist before UFE. Reports of cancer cases after UFE^81–90^ emphasize the need for preprocedure counseling regarding occult malignancy, particularly for women at higher risk for gynecologic cancer.

In 2014, the Society of Interventional Radiology (SIR) published quality improvement guidelines for UFE and highlighted that interventional radiologists should counsel patients about “the possibility of a missed diagnosis of cancer and a delay in definitive management,” referring to the American Association of Gynecologic Laparoscopists and American College of Obstetricians and Gynecologists (ACOG) statements from that year to quote leiomyosarcoma risk.^90^ Studies have estimated gynecologic cancer incidence after UFE to be 1 in 497 women in 3 years, which varied based on age.^46,88,89,91^

We do know what actual compliance with SIR's guidelines is in clinical practice in their IR community. Meanwhile, the controversy of incidentally morcellating leiomyosarcoma or having other cancer or premalignant conditions at the time of hysterectomy in 2014 had a profound effect on an entire surgical community.^86,87^ Unfortunately, media attention was one-sided highlighting the risk of undiagnosed cancer during surgery, while some advertisements for UFE continue to call it an “alternative to morcellation,” suggesting that embolizing an Leiomyosarcoma without a tissue diagnosis would be a better option.

Another factor that needs to be added to the risk calculation is ovarian cancer risk reduction with opportunistic salpingectomy during surgery and evolving understanding of the role of oophorectomy at the time of hysterectomy.^92^

#### Preoperative evaluation

The ACOG recommends that patients undergo a consultation with a gynecologist familiar with UFE to perform a thorough preoperative evaluation and counseling.^93^ Such a statement underscores the importance of “gynecology clearance” for UFE. This should include thorough counseling on options for fibroid treatment in addition to ensuring cervical cancer screening is up-to-date and endometrial biopsy is completed if indicated.

Apart from gynecology consultation, there are no recommendations for any further medical clearance for UFE. Given similar rates of complications between UFE and minimally invasive gynecologic procedures, it only seems logical that patients should be medically optimized before UFE similar to the “medical clearance” that is obtained before surgery.

#### Direct restorative remedy #4

Bring to the forefront of clinical and research discourse less frequently addressed concerns with fertility, menopause and sexual function, risk of malignancy, and pre-operative assessment.

### Reimbursement does not reflect the value of work

#### Economics of UFE

Uterine leiomyomas affect up to 70–80% of women before the age of 50.^94^ Given the lack of effective medical therapies, ∼200,000 hysterectomies and 30,000 myomectomies are performed annually in the United States, making fibroid surgery one of the most commonly performed major surgeries.^95–97^

These numbers represent a significant market share available to any entity that wants to capitalize on treatments, from pharma and medical device companies to hospitals and private practitioners. This incentivized introduction of several nonsurgical uterine-spearing procedural alternatives over the last few decades. While no accurate tracking systems exist for reporting UFE incidence, most recent estimates noted over 25,000 UFE procedures performed annually worldwide and about 14,000 in the United States.^18,62,98,99^ Relative to surgery, UFE is performed in a much small percentage of women (based on the above estimates, about 5%).

Notably, while the overall long-term cost-effectiveness of UFE is not favorable when compared with the surgery,^32^ total reimbursement in outpatient private practice settings is well-reimbursed and is profitable.^100,101^ As a result, UFE providers in a private setting often engage in effective direct marketing to patients.^102^ Gynecologic surgery and hospital-based work (where most radiologists who perform UFE are based in academic centers) remain underpaid and cover more uninsured and underinsured patients compared with “private centers,” which can collect from well-reimbursing commercial insurance. In our urban academic center, for example, such centers (which call themselves “fibroid centers”) are growing rapidly. This is one of the downstream effects of discrimination against MIGS surgeons outlined in a recent commentary; the value of surgical care delivered to women is ignored by systemic sexism, complex, and as result, patients and surgeons suffer.^103^

#### Procedural alternatives

Given the financial success of UFE, several other interventional alternatives have been recently introduced to the U.S. market: Magnetic resonance-guided focused ultrasound (MRgFUS, ExAblate™)^104^ and Ultrasound-guided RFA (Acessa™ and Sonata™).^105–107^ While promising alternatives due to their more focal nature, uptake of these in clinical practice has been slow due to barriers in reimbursement, and comparative effectiveness trials remain limited.^108^

##### Direct restorative remedy #5

Change the payment system from fee-for-service to value-based and create financial insensitive for individuals, instituions and health sytems that will prioritize delivery of high-quality care to women.

### Lack of coordination between specialties and services

#### Patient selection

While most entities agree on absolute contraindications such as pregnancy, active infection, or suspected cancer, relative contraindications are still unclear and there is little guidance on how to select an “ideal candidate.” Even in uncomplicated cases, counseling becomes complex and nuanced with limited data to rely on, let alone for more complex patients with large uterine volumes and those desiring to preserve fertility.^90^

Unfortunately, there is little interprofessional collaboration between radiologists and gynecologists in academia. Each specialty typically writes its articles and reads its journals. Historically, the limited guidelines surrounding UFE have typically been generated by the Obstetrics and Gynecology and IR groups separately, without comprehensive collaborative efforts. For example, a working group of interventional radiologists recently came up with a metric system of thresholds for outcomes and complications with hopes of helping clinicians implement UFE services^90,109^; however, this work did not include coordination of care and details of how high-quality programs should be implemented. While consensus is emerging that patient selection is a key factor,^34^ no joint coherent guidelines exist to reconcile the viewpoints of both gynecologists and radiologists and emphasize multispecialty care.

#### Create fibroid centers of excellence

The lack of coordinated delivery of care has been addressed in a few publications, but they are not reaching high visibility in either specialty. When patients are only seen by interventional radiologists, coordination with gynecologists is seen by some as nonrevenue generating, time-consuming, and burdensome.^110^ A study by Nakhaei et al. highlighted how the coordination of care between gynecologists and interventional radiologists may be critical to optimizing patient selection and outcomes.^110^ Tan et al. described a comprehensive fibroid center at their institution and resulting in improvements in individualized care.^111^ While there is a multitude of publications on multidisciplinary care programs in other domains, there is a lack of publications on multidisciplinary care for fibroids.^112,113^

Given the lack of centralized guidance and a clear need to take care of a large number of patients with fibroids, institutions on a local level are taking matters into their own hands and creating multispecialty fibroid centers to address this problem (pers. comm.).^114,115^ On a positive note, inspiring and encouraging initiatives are carried out from the “ground up” by clinicians and vary in their extent and scale. A general implementation plan can include suggested steps outlined in Table 1. While this list is extensive, it should be adopted for each specific institution. For example, a hospital with limited resources might be able to launch a UFE clinic by allowing joint clinic panels between gynecologists and interventional radiologists to occur several times a month while using existing support staff and utilizing simple and free ways to disseminate information.

**Table 1.** Outline for implementation of collaborative uterine fibroid embolization service line

• Define the problem and goals
• Identify stakeholders
○ IR clinicians
○ Gynecologic surgeons
○ Referring clinicians in the community
○ Patients
○ Administrators (gynecology, international radiology)
○ Potential support clinic and procedure staff (nurse practitioners, physician assistants, nurses)
• Perform needs assessment, address, and prioritize issues uncovered
• Create an evidence-based institutional protocol to address
○ Patient selection
○ Multidisciplinary case conference to meet on regular basis and review higher complexity cases
○ Counseling
○ Preprocedure evaluation including:
▪ Up-to-date cervical cancer screening test
▪ Endometrial sampling based on clinical presentation
▪ Appropriate imaging
▪ Medical clearance if indicated
○ Intra- and postprocedural care
○ Postprocedural follow up
○ Assessment of patient satisfaction
○ Monitoring and review of outcomes
○ The process to address complications
○ Patient education materials
○ Clinician education materials
○ Counseling checklist with sample counseling script and documentation template
○ Procedural checklist
• Define desired outcomes
○ Ease of access to care for patients and referring clinicians
○ The patient selection that met the criteria
○ Complication rates
○ Success rates
• Formulate implementation plan based on above, to include
○ Identify champions who will lead and implement
○ Identify resources
▪ Financial support for upfront and maintenance costs
▪ Salary lines
▪ Protected time for the champions and support staff
▪ Physical space
▪ Marketing
○ The physical location of the clinic where patients are seen (ideally should include both a gynecologist and interventional radiologist)
○ Outcome database
▪ Define metrics
▪ Formulate how data will be collected, analyzed, and presented
▪ Allocate resources
• Present implementation plan to leadership for feedback and approval
• Survey program during implementation and maintenance for improvement opportunities

IR, interventional radiology.

On the contrary, an institution with more resources might choose to finance the large-scale building of a full-service line with additional salary lines, staff, and a marketing campaign. It is important to note that in either case, financial success should be defined by desired outcomes rather than itemized collections by each service line. In the best-case scenario, if you get a gynecologic surgeon and an interventional radiologist to review a patient's case before seeing them, and then counsel the patient together in the same room at the same time, the patient is more likely to get a full picture rather than a disjointed one.

While we realize that the major challenge of choosing the appropriate treatment for symptomatic fibroids is a highly variable presentation for each patient and no suitable formula or calculator to fit them into, we nevertheless propose one way to approach patient selection in Table 2 based on the best available evidence. For example, a morbidly obese 45-year-old woman who is done with childbearing with a 12-week-sized fibroid uterus with mostly intramural myomas with a history of two prior laparotomies and who is concerned with surgical risk can consider UFE as a viable option.

**Table 2.** Proposed patient selection scheme for uterine fibroid embolization

Clinical factors favorable for UFE as a treatment option	Consider UFE only with caution/relative contraindications	Absolute contraindications
• Older age • Desire to avoid major surgery • Medical conditions that increase risk for surgical intervention	• Patients desiring future fertility • Pedunculated fibroids • Submucosal fibroids • Massive fibroid burden • Diminished ovarian reserve • Previous GnRHa treatment • Concerning features for atypical myomata on MRI	• Pregnancy • Known malignancy • Active infection

GnRHa, gonadotropin-releasing hormone agonist; MRI, magnetic resonance imaging; UFE, uterine fibroid embolization.

On the contrary, a 38-year-old patient who is attempting pregnancy with a 14-week-sized uterus and a 5-cm intramural myoma with a submucosal component should be counseled that UFE is a poor choice for her given the impact on her fertility and high failure rate. Likewise, a 48-year-old woman with a 28-week-sized fibroid uterus and bothersome bulk symptoms should be told that UFE is not going to provide her with adequate symptomatic relief.

##### Direct restorative remedy #6

Define meaningful patient selection criteria and create collaborative patient-centered service lines to deliver care.

## Proposed Solutions: What Needs to Happen to Deliver High-Quality Medical Care

While the solutions proposed above are a starting point for discourse, we feel that it is important to voice our concern about the delivery of medical care in our country on the large scale. Without a complete overhaul, any efforts at improvement are not likely to make a difference for most patients, and our “solutions” will end up being isolated and short-term measures even if they are successfully implemented. While this proposition seems radical and unrealistic, many social justice movements were also considered to be historical and this movement to revolutionize American health care is getting more and more momentum.^116,117^

While Victor Montori argues in his book “Why We Revolt” that health care needs to be redesigned to provide “careful and kind care,” we believe that a doctor–patient alliance and advocacy movement needs to take control of all health care to do the right thing for each patient and deliver meaningful improvements.

On a larger scale, the idea of industrial health care in which health care entities need to make money to survive is considered not only wrong but also not viable from a business standpoint. Domains such as health care, education, childcare, eldercare, and other services such as national parks are viewed as a public good, not an opportunity to make money. It is viewed as a basic infrastructure that society needs to function. Health care is a cost of doing business, not a way to make a profit. Companies see the health of their workforce as a necessity to stay completive and as a result view health care expenses as a “sunk cost,” just like the rent they pay for their office space.

One of the factors is the fee-for-service payment model. If you get paid regardless, why bother with real outcome research? Lack of prospective data collection to answer simple clinical questions? Who would pay for this? Data collection and monitoring of outcomes for surgery and UFE on a national level? Lack of allocation of money to delivery of gynecologic care while other areas end up with getting resources because of politics? None of these deeper questions can be addressed without a complete overhaul. Of note, several attempts to implement change within the existing system, the most visible of which was a Buffett-Bezos-Dimon company, Heaven Healthcare, headed by physician-activist, Atul Gawande, as its CEO,^118^ have failed miserably given the premise of needing to redesign the system rather than trying to work within the existing structure.^119^

This feeling of powerlessness to make meaningful improvements to the care our patients receive is how many, if not most, clinicians feel about their day-to-day professional lives and greatly contributes to moral injury and burnout. Surgeons quit operating, and MIGS positions are unfilled in many hospitals. In the last 5 years, six fellowship-trained gynecologic surgeons stopped operating in a 5-mile radius in New York City alone (pers. comm.). We think that all of us need to speak out and expose injustices that prevent us from doing our best for our patients. The first step would be to expose the problem while working on finding ways to do it right.

## Conclusions

In this article, we used fibroid care as an example to dissect factors and drivers of what goes on “behind the scenes” and results in failure of delivery of standard of care. We described the historical background of the barriers clinicians face in the trenches, such as the sequelae of misogyny from underfunding of research in women's health to lack of integrated approach, and poor communication. We also addressed economic disincentives to do the right thing, and we discussed potential remedies to those problems. We hope patients, clinicians, advocates, and stakeholders can benefit from this discussion and feel validated in their struggle to do their best in caring for patients.

## Abbreviations Used

ACOG: American College of Obstetricians and Gynecologists
ART: assisted reproductive technology
CI: confidence interval
GnRHa: gonadotropin-releasing hormone agonist
IR: interventional radiology
IVF: *in vitro* fertilization
MIGS: minimally invasive gynecologic surgery
MIS: minimally invasive surgery
MRgFUS: magnetic resonance imaging-guided focused ultrasound
MRI: magnetic resonance imaging
OR: odds ratio
RCT: randomized controlled trial
RFA: radiofrequency ablation
RR: relative risk
SIR: Society of Interventional Radiology
UFE: uterine fibroid embolization
UFS-QOL: Uterine Fibroid Symptom and Quality of Life Questionnaire

## References

[1] King LP. A reluctant critic: Why gynecologic surgery needs reform. Hastings Cent Rep 2019;49(3):10–13. 10.1002/hast.1001 31269250

[2] Marsh EE, Al-Hendy A, Kappus D, et al. Burden, prevalence, and treatment of uterine fibroids: A survey of U.S. women. J Womens Health (Larchmt) 2018;27(11):1359–1367. 30230950 10.1089/jwh.2018.7076 PMC6247381

[3] Eltoukhi HM, Modi MN, Weston M, et al. The health disparities of uterine fibroid tumors for African American women: A public health issue. Am J Obstet Gynecol 2014;210(3):194–199. 23942040 10.1016/j.ajog.2013.08.008 PMC3874080

[4] Laughlin-Tommaso SK, Jacoby VL, Myers ER. Disparities in fibroid incidence, prognosis, and management. Obstet Gynecol Clin North Am 2017;44(1):81–94. 28160895 10.1016/j.ogc.2016.11.007

[5] Jacoby VL, Fujimoto VY, Giudice LC, et al. Racial and ethnic disparities in benign gynecologic conditions and associated surgeries. Am J Obstet Gynecol 2010;202(6):514–521. 20430357 10.1016/j.ajog.2010.02.039 PMC4625911

[6] Anderson CTM. Taking Back Our Voices—#HumanityIsOurLane. N Engl J Med 2020;383(17):1609–1611. 32603037 10.1056/NEJMp2021291

[7] Traylor J, Chaudhari A, Tsai S, et al. Patient-reported outcome measures in benign gynecologic surgery: Updates and selected tools. Curr Opin Obstet Gynecol 2019;31(4):259–266. 30973376 10.1097/GCO.0000000000000544

[8] Aseltine R, Carlson K, Fowler F, et al. Comparing prospective and retrospective measures of treatment outcomes. Med Care 1995;33:AS67–AS76. 7536868

[9] Carlson KJ, Miller BA, Fowler FJ Jr. The Maine Women's Health Study: I. Outcomes of hysterectomy. Obstet Gynecol 1994;83(4):556–565. 8134066 10.1097/00006250-199404000-00012

[10] Carlson KJ, Miller BA, Fowler FJ Jr. The Maine Women's Health Study: II. Outcomes of nonsurgical management of leiomyomas, abnormal bleeding, and chronic pelvic pain. Obstet Gynecol 1994;83(4):566–572. 8134067 10.1097/00006250-199404000-00013

[11] Spies JB, Coyne K, Guaou Guaou N, et al. The UFS-QOL, a new disease-specific symptom and health-related quality of life questionnaire for leiomyomata. Obstet Gynecol 2002;99(2):290–300. 11814511 10.1016/s0029-7844(01)01702-1

[12] Agarwal SK, Chapron C, Giudice LC, et al. Clinical diagnosis of endometriosis: A call to action. Am J Obstet Gynecol 2019;220(4):354.e1–354.e12. 10.1016/j.ajog.2018.12.039 30625295

[13] Al-Hendy A, Myers ER, Stewart E. Uterine fibroids: Burden and unmet medical need. Semin Reprod Med 2017;35(6):473–480. 29100234 10.1055/s-0037-1607264 PMC6193285

[14] Marsh EE, Chibber S, Saad W. Patient-centered care and uterine fibroids. Semin Reprod Med 2017;35(6):560–564. 29100243 10.1055/s-0037-1607267

[15] Ravina JH, Herbreteau D, Ciraru-Vigneron N, et al. Arterial embolisation to treat uterine myomata. Lancet 1995;346(8976):671–672. 7544859 10.1016/s0140-6736(95)92282-2

[16] Spies JB, Myers ER, Worthington-Kirsch R, et al. The FIBROID Registry: Symptom and quality-of-life status 1 year after therapy. Obstet Gynecol 2005;106(6):1309–1318. 16319257 10.1097/01.AOG.0000188386.53878.49

[17] Worthington-Kirsch R, Spies JB, Myers ER, et al. The Fibroid Registry for outcomes data (FIBROID) for uterine embolization: Short-term outcomes. Obstet Gynecol 2005;106(1):52–59. 15994617 10.1097/01.AOG.0000165828.68787.a9

[18] Goodwin SC, Spies JB, Worthington-Kirsch R, et al. Uterine artery embolization for treatment of leiomyomata: Long-term outcomes from the FIBROID Registry. Obstet Gynecol 2008;111(1):22–33. 18165389 10.1097/01.AOG.0000296526.71749.c9

[19] Hirst A, Dutton S, Wu O, et al. A multi-centre retrospective cohort study comparing the efficacy, safety and cost-effectiveness of hysterectomy and uterine artery embolisation for the treatment of symptomatic uterine fibroids. The HOPEFUL study. Health Technol Assess 2008;12(5):1–248, iii. 10.3310/hta12050 18331704

[20] de Bruijn AM, Ankum WM, Reekers JA, et al. Uterine artery embolization vs hysterectomy in the treatment of symptomatic uterine fibroids: 10-Year outcomes from the randomized EMMY trial. Am J Obstet Gynecol 2016;215(6):745.e1–745.e12. 10.1016/j.ajog.2016.06.051 27393268

[21] Scheurig-Muenkler C, Koesters C, Powerski MJ, et al. Clinical long-term outcome after uterine artery embolization: Sustained symptom control and improvement of quality of life. J Vasc Interv Radiol 2013;24(6):765–771. 23582992 10.1016/j.jvir.2013.02.018

[22] Villanueva C, Talwar A, Doyle M. Improving informed consent in cardiac surgery by enhancing preoperative education. Patient Educ Couns 2018;101(12):2047–2053. 29937111 10.1016/j.pec.2018.06.008

[23] Reed SD, Newton KM, Thompson LB, et al. The incidence of repeat uterine surgery following myomectomy. J Womens Health (Larchmt) 2006;15(9):1046–1052. 17125423 10.1089/jwh.2006.15.1046

[24] Davis MR, Soliman AM, Castelli-Haley J, et al. Reintervention rates after myomectomy, endometrial ablation, and uterine artery embolization for patients with uterine fibroids. J Womens Health (Larchmt) 2018;27(10):1204–1214. 30085898 10.1089/jwh.2017.6752 PMC6205049

[25] Doridot V, Dubuisson JB, Chapron C, et al. Recurrence of leiomyomata after laparoscopic myomectomy. J Am Assoc Gynecol Laparosc 2001;8(4):495–500. 11677326 10.1016/s1074-3804(05)60610-x

[26] van der Kooij SM, Ankum WM, Hehenkamp WJ. Review of nonsurgical/minimally invasive treatments for uterine fibroids. Curr Opin Obstet Gynecol 2012;24(6):368–375. 23014141 10.1097/GCO.0b013e328359f10a

[27] Manyonda IT, Bratby M, Horst JS, et al. Uterine artery embolization versus myomectomy: Impact on quality of life—Results of the FUME (Fibroids of the Uterus: Myomectomy versus Embolization) trial. Cardiovasc Intervent Radiol 2012;35(3):530–536. 21773858 10.1007/s00270-011-0228-5

[28] Jun F, Yamin L, Xinli X, et al. Uterine artery embolization versus surgery for symptomatic uterine fibroids: A randomized controlled trial and a meta-analysis of the literature. Arch Gynecol Obstet 2012;285(5):1407–1413. 22048783 10.1007/s00404-011-2065-9

[29] Mara M, Fucikova Z, Maskova J, et al. Uterine fibroid embolization versus myomectomy in women wishing to preserve fertility: preliminary results of a randomized controlled trial. Eur J Obstet Gynecol Reprod Biol 2006;126(2):226–233. 16293363 10.1016/j.ejogrb.2005.10.008

[30] Mara M, Maskova J, Fucikova Z, et al. Midterm clinical and first reproductive results of a randomized controlled trial comparing uterine fibroid embolization and myomectomy. Cardiovasc Intervent Radiol 2008;31(1):73–85. 17943348 10.1007/s00270-007-9195-2 PMC2700241

[31] Pinto I, Chimeno P, Romo A, et al. Uterine fibroids: Uterine artery embolization versus abdominal hysterectomy for treatment—A prospective, randomized, and controlled clinical trial. Radiology 2003;226(2):425–431. 12563136 10.1148/radiol.2262011716

[32] Moss JG, Cooper KG, Khaund A, et al. Randomised comparison of uterine artery embolisation (UAE) with surgical treatment in patients with symptomatic uterine fibroids (REST trial): 5-Year results. BJOG 2011;118(8):936–944. 21481151 10.1111/j.1471-0528.2011.02952.x

[33] Ruuskanen A, Hippelainen M, Sipola P, et al. Uterine artery embolisation versus hysterectomy for leiomyomas: Primary and 2-year follow-up results of a randomised prospective clinical trial. Eur Radiol 2010;20(10):2524–2532. 20526776 10.1007/s00330-010-1829-0

[34] Gupta JK, Sinha A, Lumsden MA, et al. Uterine artery embolization for symptomatic uterine fibroids. Cochrane Database Syst Rev 2014;2014(12):CD005073. 10.1002/14651858.CD005073.pub4 PMC11285296 25541260

[35] Fonseca MCM, Castro R, Machado M, et al. Uterine artery embolization and surgical methods for the treatment of symptomatic uterine leiomyomas: A systemic review and meta-analysis followed by indirect treatment comparison. Clin Ther 2017;39(7):1438–1455.e2. 28641997 10.1016/j.clinthera.2017.05.346

[36] Sandberg EM, Tummers F, Cohen SL, et al. Reintervention risk and quality of life outcomes after uterine-sparing interventions for fibroids: A systematic review and meta-analysis. Fertil Steril 2018;109(4):698–707.e1. 29653718 10.1016/j.fertnstert.2017.11.033

[37] Hartmann KE FC, Surawicz T, Krishnaswami S, et al. Management of Uterine Fibroids. Comparative Effectiveness Review No. 195. Agency for Healthcare Research and Quality: AHRQ Publication No. 17(18)-EHC028-EF: Rockville, MD; 2017 [Prepared by the Vanderbilt Evidence-based Practice Center under Contract No. 290-2015-00003-I].

[38] Havryliuk Y, Setton R, Carlow JJ, et al. Symptomatic fibroid management: Systematic review of the literature. JSLS 2017;21(3):e2017.00041. 10.4293/JSLS.2017.00041 PMC5600131 28951653

[39] El Shamy T, Amer SAK, Mohamed AA, et al. The impact of uterine artery embolization on ovarian reserve: A systematic review and meta-analysis. Acta Obstet Gynecol Scand 2020;99(1):16–23. 31370100 10.1111/aogs.13698

[40] Stewart EA, Lytle BL, Thomas L, et al. The Comparing Options for Management: PAtient-centered REsults for Uterine Fibroids (COMPARE-UF) registry: Rationale and design. Am J Obstet Gynecol 2018;219(1):95.e1–95.e10. 10.1016/j.ajog.2018.05.004 PMC8889489 29750955

[41] Wallace K, Stewart EA, Wise LA, et al. Anxiety, depression, and quality of life after procedural intervention for uterine fibroids. J Womens Health (Larchmt) 2022;31(3):415–424. 34101502 10.1089/jwh.2020.8915 PMC8972021

[42] Wallace K, Zhang S, Thomas L, et al. Comparative effectiveness of hysterectomy versus myomectomy on one-year health-related quality of life in women with uterine fibroids. Fertil Steril 2020;113(3):618–626. 32192594 10.1016/j.fertnstert.2019.10.028

[43] van der Kooij SM, Bipat S, Hehenkamp WJ, et al. Uterine artery embolization versus surgery in the treatment of symptomatic fibroids: A systematic review and metaanalysis. Am J Obstet Gynecol 2011;205(4):317.e1–e18. 10.1016/j.ajog.2011.03.016 21641570

[44] Lai AC, Goodwin SC, Bonilla SM, et al. Sexual dysfunction after uterine artery embolization. J Vasc Interv Radiol 2000;11(6):755–758. 10877421 10.1016/s1051-0443(07)61635-2

[45] Clarke-Pearson DL, Geller EJ. Complications of hysterectomy. Obstet Gynecol 2013;121(3):654–673. 23635631 10.1097/AOG.0b013e3182841594

[46] Spies JB, Spector A, Roth AR, et al. Complications after uterine artery embolization for leiomyomas. Obstet Gynecol 2002;100(5 Pt 1):873–880. 12423844 10.1016/s0029-7844(02)02341-4

[47] Armstrong AA, Kroener L, Brower M, et al. Analysis of reported adverse events with uterine artery embolization for leiomyomas. J Minim Invasive Gynecol 2019;26(4):667–670.e1. 30016750 10.1016/j.jmig.2018.07.006

[48] Schirf BE, Vogelzang RL, Chrisman HB. Complications of uterine fibroid embolization. Semin Intervent Radiol 2006;23(2):143–149. 21326757 10.1055/s-2006-941444 PMC3036365

[49] Martin J, Bhanot K, Athreya S. Complications and reinterventions in uterine artery embolization for symptomatic uterine fibroids: A literature review and meta analysis. Cardiovasc Intervent Radiol 2013;36(2):395–402. 23152035 10.1007/s00270-012-0505-y

[50] Volkers NA, Hehenkamp WJ, Birnie E, et al. Uterine artery embolization versus hysterectomy in the treatment of symptomatic uterine fibroids: 2 years' outcome from the randomized EMMY trial. Am J Obstet Gynecol 2007;196(6):519.e1–519.e11. 10.1016/j.ajog.2007.02.029 17547877

[51] Shlansky-Goldberg RD, Coryell L, Stavropoulos SW, et al. Outcomes following fibroid expulsion after uterine artery embolization. J Vasc Interv Radiol 2011;22(11):1586–1593. 22024118 10.1016/j.jvir.2011.08.004

[52] Pinto E, Trovao A, Leitao S, et al. Conservative laparoscopic approach to a perforated pyomyoma after uterine artery embolization. J Minim Invasive Gynecol 2012;19(6):775–779. 23084686 10.1016/j.jmig.2012.07.001

[53] Sterling L, Boutet M, Colak E, et al. Fibroid infected with *Escherichia coli* requiring surgical removal following uterine artery embolization. J Obstet Gynaecol Can 2013;35(9):823–826. 24099448 10.1016/S1701-2163(15)30839-2

[54] Dewdney SB, Mani NB, Zuckerman DA, et al. Uteroenteric fistula after uterine artery embolization. Obstet Gynecol 2011;118(2 Pt 2):434–436. 21768845 10.1097/AOG.0b013e31821082a3

[55] Virmani V, Fasih N, Rakhra K. Intraluminal bowel obstruction by a detached fibroid—An extremely unusual complication of uterine artery embolization. Clin Radiol 2011;66(8):795–797. 21571263 10.1016/j.crad.2011.03.015

[56] Acharya J, Bancroft K, Lay J. Perforation of transverse colon: A catastrophic complication of uterine artery embolization for fibroids. Cardiovasc Intervent Radiol 2012;35(6):1524–1527. 22278665 10.1007/s00270-012-0349-5

[57] Mallick R, Okojie A, Ajala T. Rectal perforation: An unusual complication of uterine artery embolisation. J Obstet Gynaecol 2016;36(7):867–868. 27184776 10.1080/01443615.2016.1180506

[58] Yeaton-Massey A, Loring M, Chetty S, et al. Uterine rupture after uterine artery embolization for symptomatic leiomyomas. Obstet Gynecol 2014;123(2 Pt 2 Suppl 2):418–420. 24413250 10.1097/AOG.0b013e3182a46df9

[59] Glaser LM, Milad MP. Bowel and bladder injury repair and follow-up after gynecologic surgery. Obstet Gynecol 2019;133(2):313–322. 30633149 10.1097/AOG.0000000000003067

[60] Stany MP, Farley JH. Complications of gynecologic surgery. Surg Clin North Am 2008;88(2):343–359, vii. 18381117 10.1016/j.suc.2007.12.004

[61] Temkin SM, Noursi S, Regensteiner JG, et al. Perspectives from advancing National Institutes of Health research to inform and improve the health of women: A conference summary. Obstet Gynecol 2022;140(1):10–19. 35849451 10.1097/AOG.0000000000004821 PMC9205296

[62] Silberzweig JE, Powell DK, Matsumoto AH, et al. Management of uterine fibroids: A focus on uterine-sparing interventional techniques. Radiology 2016;280(3):675–692. 27533290 10.1148/radiol.2016141693

[63] Ludwig PE, Huff TJ, Shanahan MM, et al. Pregnancy success and outcomes after uterine fibroid embolization: Updated review of published literature. Br J Radiol 2020;93(1105):20190551. 31573326 10.1259/bjr.20190551 PMC6948071

[64] Karlsen K, Hrobjartsson A, Korsholm M, et al. Fertility after uterine artery embolization of fibroids: A systematic review. Arch Gynecol Obstet 2018;297(1):13–25. 29052017 10.1007/s00404-017-4566-7

[65] Gupta JK, Sinha A, Lumsden MA, et al. Uterine artery embolization for symptomatic uterine fibroids. Cochrane Database Syst Rev 2012;2012(5):CD005073. 10.1002/14651858.CD005073.pub3 22592701

[66] Homer H, Saridogan E. Uterine artery embolization for fibroids is associated with an increased risk of miscarriage. Fertil Steril 2010;94(1):324–330. 19361799 10.1016/j.fertnstert.2009.02.069

[67] Pron G, Mocarski E, Bennett J, et al. Pregnancy after uterine artery embolization for leiomyomata: The Ontario multicenter trial. Obstet Gynecol 2005;105(1):67–76. 15625144 10.1097/01.AOG.0000149156.07061.1f

[68] Walker WJ, McDowell SJ. Pregnancy after uterine artery embolization for leiomyomata: A series of 56 completed pregnancies. Am J Obstet Gynecol 2006;195(5):1266–1271. 16796984 10.1016/j.ajog.2006.04.011

[69] Chrisman HB, Saker MB, Ryu RK, et al. The impact of uterine fibroid embolization on resumption of menses and ovarian function. J Vasc Interv Radiol 2000;11(6):699–703. 10877413 10.1016/s1051-0443(07)61627-3

[70] Kaump GR, Spies JB. The impact of uterine artery embolization on ovarian function. J Vasc Interv Radiol 2013;24(4):459–467. 23384832 10.1016/j.jvir.2012.12.002

[71] Kim CW, Shim HS, Jang H, et al. The effects of uterine artery embolization on ovarian reserve. Eur J Obstet Gynecol Reprod Biol 2016;206:172–176. 27697621 10.1016/j.ejogrb.2016.09.001

[72] Walker WJ, Pelage JP. Uterine artery embolisation for symptomatic fibroids: Clinical results in 400 women with imaging follow up. BJOG 2002;109(11):1262–1272. 12452465 10.1046/j.1471-0528.2002.01449.x

[73] Hehenkamp WJ, Volkers NA, Broekmans FJ, et al. Loss of ovarian reserve after uterine artery embolization: A randomized comparison with hysterectomy. Hum Reprod 2007;22(7):1996–2005. 17582145 10.1093/humrep/dem105

[74] Rashid S, Khaund A, Murray LS, et al. The effects of uterine artery embolisation and surgical treatment on ovarian function in women with uterine fibroids. BJOG 2010;117(8):985–989. 20465558 10.1111/j.1471-0528.2010.02579.x

[75] Kovacsik HV, Herbreteau D, Bommart S, et al. Evaluation of changes in sexual function related to uterine fibroid embolization (UFE): Results of the EFUZEN study. Cardiovasc Intervent Radiol 2017;40(8):1169–1175. 28321542 10.1007/s00270-017-1615-3

[76] Bossick AS, Sangha R, Olden H, et al. Identifying what matters to hysterectomy patients: Postsurgery perceptions, beliefs, and experiences. J Patient Cent Res Rev 2018;5(2):167–175. 29774227 10.17294/2330-0698.1581 PMC5953196

[77] Roovers JP, van der Bom JG, van der Vaart CH, et al. Hysterectomy and sexual wellbeing: prospective observational study of vaginal hysterectomy, subtotal abdominal hysterectomy, and total abdominal hysterectomy. BMJ 2003;327(7418):774–778. 14525872 10.1136/bmj.327.7418.774 PMC214074

[78] Radosa JC, Meyberg-Solomayer G, Kastl C, et al. Influences of different hysterectomy techniques on patients' postoperative sexual function and quality of life. J Sex Med 2014;11(9):2342–2350. 25042204 10.1111/jsm.12623

[79] Berlit S, Tuschy B, Wuhrer A, et al. Sexual functioning after total versus subtotal laparoscopic hysterectomy. Arch Gynecol Obstet 2018;298(2):337–344. 29948170 10.1007/s00404-018-4812-7

[80] Dedden SJ, van Ditshuizen MAE, Theunissen M, et al. Hysterectomy and sexual (dys)function: An analysis of sexual dysfunction after hysterectomy and a search for predictive factors. Eur J Obstet Gynecol Reprod Biol 2020;247:80–84. 32078980 10.1016/j.ejogrb.2020.01.047

[81] Cohen SL, Hariton E, Afshar Y, et al. Updates in uterine fibroid tissue extraction. Curr Opin Obstet Gynecol 2016;28(4):277–282. 27253236 10.1097/GCO.0000000000000280

[82] Siedhoff MT, Cohen SL. Tissue extraction techniques for leiomyomas and uteri during minimally invasive surgery. Obstet Gynecol 2017;130(6):1251–1260. 29112659 10.1097/AOG.0000000000002334

[83] ACOG Committee Opinion No. 770: Uterine morcellation for presumed leiomyomas. Obstet Gynecol 2019;133(3):e238–e248. 30801477 10.1097/AOG.0000000000003126

[84] Tissue Extraction Task Force Members. Morcellation during uterine tissue extraction: An update. J Minim Invasive Gynecol 2018;25(4):543–550. 29581072 10.1016/j.jmig.2018.03.010

[85] AAGL Advancing Minimally Invasive Gynecology Worldwide. AAGL practice report: Morcellation during uterine tissue extraction. J Minim Invasive Gynecol 2014;21(4):517–530. 24865630 10.1016/j.jmig.2014.05.010

[86] Wright JD, Tergas AI, Cui R, et al. Use of electric power morcellation and prevalence of underlying cancer in women who undergo myomectomy. JAMA Oncol 2015;1(1):69–77. 26182307 10.1001/jamaoncol.2014.206

[87] Wright JD, Tergas AI, Burke WM, et al. Uterine pathology in women undergoing minimally invasive hysterectomy using morcellation. JAMA 2014;312(12):1253–1255. 25051495 10.1001/jama.2014.9005 PMC4231432

[88] Buzaglo K, Bruchim I, Lau SK, et al. Sarcoma post-embolization for presumed uterine fibroids. Gynecol Oncol 2008;108(1):244–247. 17950451 10.1016/j.ygyno.2007.09.012

[89] Choi JI, Lee HJ, Shin YJ, et al. Rapid enlargement of endometrial stromal sarcoma after uterine fibroid embolization for presumed adenomyosis: A case report and literature review. Eur J Gynaecol Oncol 2016;37(6):876–881. 29943942

[90] Dariushnia SR, Nikolic B, Stokes LS, et al. Quality improvement guidelines for uterine artery embolization for symptomatic leiomyomata. J Vasc Interv Radiol 2014;25(11):1737–1747. 25442136 10.1016/j.jvir.2014.08.029

[91] Bronico JVR, Matthews B, Perkins RB, et al. Incidence of gynecologic cancers in women after uterine artery embolization. J Minim Invasive Gynecol 2021;28(6):1231–1236. 33115685 10.1016/j.jmig.2020.10.015 PMC8609291

[92] Rush SK, Ma X, Newton MA, et al. A revised markov model evaluating oophorectomy at the time of hysterectomy for benign indication: Age 65 years revisited. Obstet Gynecol 2022;139(5):735–744; doi: 10.1097/AOG.0000000000004732 35576331 PMC9015029

[93] ACOG Practice Bulletin. Alternatives to hysterectomy in the management of leiomyomas. Obstet Gynecol 2008;112(2 Pt 1):387–400. 18669742 10.1097/AOG.0b013e318183fbab

[94] Baird DD, Dunson DB, Hill MC, et al. High cumulative incidence of uterine leiomyoma in black and white women: Ultrasound evidence. Am J Obstet Gynecol 2003;188(1):100–107. 12548202 10.1067/mob.2003.99

[95] Wu JM, Wechter ME, Geller EJ, et al. Hysterectomy rates in the United States, 2003. Obstet Gynecol 2007;110(5):1091–1095. 17978124 10.1097/01.AOG.0000285997.38553.4b

[96] Wechter ME, Stewart EA, Myers ER, et al. Leiomyoma-related hospitalization and surgery: Prevalence and predicted growth based on population trends. Am J Obstet Gynecol 2011;205(5):492.e1–e5. 10.1016/j.ajog.2011.07.008 PMC3746963 22035951

[97] Wright JD, Huang Y, Li AH, et al. Nationwide estimates of annual inpatient and outpatient hysterectomies performed in the United States. Obstet Gynecol 2022;139(3):446–448. 35115477 10.1097/AOG.0000000000004679

[98] Glass Lewis M, Ekundayo OT. Cost and distribution of hysterectomy and uterine artery embolization in the United States: Regional/rural/urban disparities. Med Sci (Basel) 2017;5(2):10. 29099026 10.3390/medsci5020010 PMC5635782

[99] You JH, Sahota DS, Yuen PM. Uterine artery embolization, hysterectomy, or myomectomy for symptomatic uterine fibroids: A cost-utility analysis. Fertil Steril 2009;91(2):580–588. 18295216 10.1016/j.fertnstert.2007.11.078

[100] Baker CM, Winkel CA, Subramanian S, et al. Estimated costs for uterine artery embolization and abdominal myomectomy for uterine leiomyomata: A comparative study at a single institution. J Vasc Interv Radiol 2002;13(12):1207–1210. 12471183 10.1016/s1051-0443(07)61966-6

[101] Goldberg J, Bussard A, McNeil J, et al. Cost and reimbursement for three fibroid treatments: Abdominal hysterectomy, abdominal myomectomy, and uterine fibroid embolization. Cardiovasc Intervent Radiol 2007;30(1):54–58. 17031734 10.1007/s00270-005-0369-5

[102] Chrisman HB, Basu PA, Omary RA. The positive effect of targeted marketing on an existing uterine fibroid embolization practice. J Vasc Interv Radiol 2006;17(3):577–581. 16567685 10.1097/01.rvi.0000204854.35429.eb

[103] Watson KL, King LP. Double discrimination, the pay gap in gynecologic surgery, and its association with quality of care. Obstet Gynecol 2021;137(4):657–661. 33706362 10.1097/AOG.0000000000004309

[104] Masciocchi C, Arrigoni F, Ferrari F, et al. Uterine fibroid therapy using interventional radiology mini-invasive treatments: Current perspective. Med Oncol 2017;34(4):52. 28236104 10.1007/s12032-017-0906-5

[105] Chudnoff S, Guido R, Roy K, et al. Ultrasound-guided transcervical ablation of uterine leiomyomas. Obstet Gynecol 2019;133(1):13–22. 30531573 10.1097/AOG.0000000000003032

[106] Bradley LD, Pasic RP, Miller LE. Clinical performance of radiofrequency ablation for treatment of uterine fibroids: Systematic review and meta-analysis of prospective studies. J Laparoendosc Adv Surg Tech A 2019;29(12):1507–1517. 31702440 10.1089/lap.2019.0550 PMC7387230

[107] Braun KM, Sheridan M, Latif EZ, et al. Surgeons' early experience with the Acessa procedure: Gaining proficiency with new technology. Int J Womens Health 2016;8:669–675. 27920582 10.2147/IJWH.S119265 PMC5126001

[108] Froeling V, Meckelburg K, Schreiter NF, et al. Outcome of uterine artery embolization versus MR-guided high-intensity focused ultrasound treatment for uterine fibroids: Long-term results. Eur J Radiol 2013;82(12):2265–2269. 24075785 10.1016/j.ejrad.2013.08.045

[109] Knuttinen MG, Stark G, Hohenwalter EJ, et al. ACR Appropriateness Criteria((R)) radiologic management of uterine leiomyomas. J Am Coll Radiol 2018;15(5S):S160–S170. 29724419 10.1016/j.jacr.2018.03.010

[110] Nakhaei M, Mojtahedi A, Faintuch S, et al. Transradial and transfemoral uterine fibroid embolization comparative study: Technical and clinical outcomes. J Vasc Interv Radiol 2020;31(1):123–129. 31771890 10.1016/j.jvir.2019.08.016

[111] Tan N, McClure TD, Tarnay C, et al. Women seeking second opinion for symptomatic uterine leiomyoma: Role of comprehensive fibroid center. J Ther Ultrasound 2014;2:3. 25512867 10.1186/2050-5736-2-3 PMC4265989

[112] Jessica Opoku-Anane MSO, Lager J, Felicia Lester JC, et al. The development of a comprehensive multidisciplinary endometriosis and chronic pelvic pain center. J Endometr Pelvic Pain Disord 2020;12(1):3–9; doi: 10.1177/2284026519899015

[113] Chen I, Money D, Yong P, et al. An evaluation model for a multidisciplinary chronic pelvic pain clinic: Application of the RE-AIM framework. J Obstet Gynaecol Can 2015;37(9):804–809. 26605450 10.1016/S1701-2163(15)30151-1

[114] Nassiri N, Balica A, Cirillo-Penn NC, et al. An academic tertiary referral center's experience with a vascular surgery-based uterine artery embolization program. Ann Vasc Surg 2018;52:90–95. 29777846 10.1016/j.avsg.2018.03.008

[115] Zurawin RK, Fischer JH, 2nd, Amir L. The effect of a gynecologist-interventional radiologist relationship on selection of treatment modality for the patient with uterine myoma. J Minim Invasive Gynecol 2010;17(2):214–221. 20226411 10.1016/j.jmig.2009.12.015

[116] NPR (Natinal Public Radio) Hidden Brain Podcast: Slaying the Fee-for-Service Monster of American Healthcare; Aired on Septemer 7, 2020. Downloaded on October 24, 2020. Available from: https://www.npr.org/transcripts/908728981 [Last accessed: July 24, 2023].

[117] Why We Revolt: The Patient Revolution. Available from: https://www.patientrevolution.org/story [Last accessed: July 24, 2023].

[118] Buffett B, Dimon appoint Dr. Atul Gawande as CEO of their newly formed health-care company. CNC news; Published June 20, 2018. Downloaded October 24, 2020. Available from: https://www.cnbc.com/2018/06/20/buffett-bezos-dimon-appoint-dr-atul-gawande-as-ceo-of-their-newly-formed-health-care-company.html [Last accessed: July 24, 2023].

[119] The Amazon-Berkshire-JPMorgan Health Venture Fails to Disrupt. Bloomberg News. May 21 DoO; 2020. Available from: https://www.bloomberg.com/news/articles/2020-05-21/bezos-buffett-dimon-joint-venture-fails-to-disrupt-health-care [Last accessed: July 24, 2023].

